# Superior Treg-Expanding Properties of a Novel Dual-Acting Cytokine Fusion Protein

**DOI:** 10.3389/fphar.2019.01490

**Published:** 2019-12-18

**Authors:** Tanja Padutsch, Maksim Sendetski, Carina Huber, Nathalie Peters, Klaus Pfizenmaier, John R. Bethea, Roland E. Kontermann, Roman Fischer

**Affiliations:** ^1^ Institute of Cell Biology and Immunology, University of Stuttgart, Stuttgart, Germany; ^2^ Stuttgart Research Center Systems Biology, University of Stuttgart, Stuttgart, Germany; ^3^ Department of Biology, Drexel University, Philadelphia, PA, United States

**Keywords:** Tregs, cytokine fusion proteins, autoimmune disease, inflammation, TNFR2, TNF, IL-2, genetic engineering

## Abstract

Autoimmune diseases are caused by uncontrolled endogenous immune responses against healthy cells. They may develop due to an impaired function of regulatory T cells (Tregs), which normally suppress self-specific effector immune cells. Interleukin 2 (IL-2) and tumor necrosis factor (TNF) have been identified as key players that promote expansion, function, and stability of Tregs. *In vivo*, both low-dose IL-2 therapy and TNF receptor 2 (TNFR2) agonism were shown to expand Tregs and alleviate autoimmunity. We here designed a novel dimeric dual-acting fusion cytokine, where mouse IL-2 is genetically linked to a TNFR2-selective single-chain TNF mutein (IL2-EHD2-sc-mTNF_R2_). IL2-EHD2-sc-mTNF_R2_ showed high affinity to TNFR2 and efficiently activated IL-2 and TNFR2-selective signaling pathways. Further, IL2-EHD2-sc-mTNF_R2_ promoted superior Treg expansion, with both the IL-2 and the TNFR2 agonist (sc-mTNF_R2_) component necessary for this biological response. Ultimately, we propose that IL2-EHD2-sc-mTNF_R2_ is a dual-acting cytokine that efficiently promotes Treg expansion and might have a superior therapeutic window than conventional IL-2-based drugs.

## Introduction

Autoimmune diseases are typically caused by endogenous immune responses against healthy cells. To avoid immune responses against self-antigens, most self-reactive T cells are eliminated during T cell development in the thymus. During this selection process potentially harmful, autoreactive T cells with effector activity are released from the thymus. However, these self-specific T cells do usually not induce autoimmune diseases because, in a healthy immune system, they are suppressed in the periphery by regulatory T cells (Tregs) (Sakaguchi et al., 2012; Klatzmann and Abbas, 2015). Most autoimmune diseases display defects in either the number or function of Tregs in the peripheral blood or the site of inflammation (Dominguez-Villar and Hafler, 2018). Further, genetic or pharmacological disruption of Treg-mediated immune control resulted in development of autoimmune disease in humans and mice (Klatzmann and Abbas, 2015). Therefore, strategies to restore and/or increase Treg function are currently developed and evaluated for clinical use.

It was well established that the cytokine interleukin-2 (IL-2) is an autocrine survival and proliferation signal for T cells in general, and considered to be the major differentiation signal for naive T cells into effector T cells (Robb, 1984). However, subsequent studies showed that the dominant role of IL-2 is the maintenance of Treg cells (Nelson, 2004), and not the development of effector and memory T cells, since loss of IL-2 signaling led to a break-down of immune tolerance and homeostasis rather than to immunodeficiency (Klatzmann and Abbas, 2015). This was confirmed by studies showing that IL-2 is a key mediator of Treg differentiation, survival and function (Liao et al., 2013; Klatzmann and Abbas, 2015). IL-2 binds to the IL-2 receptor (IL-2R), which is generated by different combinations of three polypeptide chains, IL-2Rα (CD25), IL-2Rβ (CD122), and IL-2Rγ (CD132). Whereas CD25 alone binds IL-2 with low affinity, the combination of CD122 and CD132 together form a complex that binds IL-2 with intermediate affinity, and the combination of all three polypeptide chains forms the high-affinity IL-2R. The high-affinity trimeric IL-2R is constitutively expressed by Tregs, whereas other subpopulations of T cells only acquire CD25 upon activation (Liao et al., 2013).

Due to the high affinity of the trimeric IL-2R on Tregs they respond to 10-100-fold lower concentrations of IL-2 than T effector cells or other low/intermediate-affinity IL-2R expressing cells (Klatzmann and Abbas, 2015). Therefore, low-dose IL-2 therapies, which allow to expand Tregs while avoiding IL-2-induced clonal expansion or activation of T effector cells, are currently under clinical investigation for the treatment of autoimmune diseases.

Next to IL-2, TNFR2 has been recently identified as an important regulator of Treg function. TNFR2 promotes expansion of mouse (Chen et al., 2007) and human (Chen et al., 2010) Tregs and stabilizes Tregs in an inflammatory environment (Chen et al., 2013). Interestingly, expression of TNFR2 defines a maximally suppressive subset of mouse Tregs (Chen et al., 2008), indicating that TNFR2 activation regulates suppressive activity of Tregs. Indeed, it was shown that exogenous activation of TNFR2 leads to expansion of Tregs, alleviates arthritic disease and protects from acute graft versus host disease (Chopra et al., 2016; Fischer et al., 2018; Lamontain et al., 2018). Our published data indicate that presence of IL-2 dramatically increases TNFR2-dependent Treg expansion *in vitro* (Fischer et al., 2017; Fischer et al., 2018). Because of the apparent requirement of a combined activation of IL-2 and TNFR2 signal pathways we have developed a novel, dual-acting cytokine fusion protein, where IL-2 is fused by genetic engineering to a TNFR2-selective TNF mutein. We here present data showing that this fusion protein is efficiently activating both IL-2 and TNFR2 signaling pathways and which leads to superior expansion of Tregs.

## Materials and Methods

### Materials

The anti-TNF (HP8001) antibody was from Hycult Biotech (Uden, The Netherlands). The anti-CD3 (clone 17A2) and anti-CD28 (#794716) antibodies were from R&D Systems (Wiesbaden-Nordenstadt, Germany). Fluorescence-labeled antibodies against CD3 (clone REA641), CD4 (clone REA604), CD25 (clone 7D4), FoxP3 (clone 3G3) and IFNγ (clone REA638) were from Miltenyi Biotech (Bergisch-Gladbach, Germany). Horseradish peroxidase (HRP)-labeled antimouse IgG antibodies were purchased by Jackson ImmunoResearch Laboratories (Suffolk, UK). Recombinant interleukin 2 (IL-2) was purchased by Immunotools (Friesoythe, Germany). Actinomycin D and 3-(4,5-dimethyl-2-thiazolyl)-2,5-diphenyl-2H-tetrazolium bromide (MTT) were from Sigma-Aldrich and 3,30,5,50-tetramethylbenzidine (TMB) substrate was purchased from Biolegend (San Diego, CA). All other chemicals were of analytical grade. Animal care was carried out in accordance with EU guidelines.

### Production and Purification of Fusion Proteins

Recombinant proteins were produced and purified as described previously (Dong et al., 2016). Briefly, HEK293-6E cells were grown in F17 medium (Life Technologies, Darmstadt, Germany), and transiently transfected with expression constructs of TNF muteins using polyethyleneimine (Sigma). On the next day, Tryptone N1 (Organotechnie, TekniScience, Terrebonne, Canada) was added to the cell culture and cells were cultivated for additional 4 days. Then, supernatant was collected, and recombinant proteins were purified by immobilized metal ion chromatography (IMAC). For this purpose, supernatant was loaded onto a column containing Ni-NTA agarose (Macherey-Nagel, Düren, Germany) and unbound proteins were washed away using IMAC wash buffer (50 mM sodium-phosphate-buffer). Bound proteins were eluted with IMAC elution buffer (50 mM sodium-phosphate-buffer, 250 mM imidazole) and dialyzed (cut-off 4-6 kDa, Roth, Karlsruhe, Germany) against PBS overnight at 4°C. Finally, eluted proteins were purified by size exclusion chromatography (SEC). Protein concentration was determined by measuring the absorbance at 280 nm. For Coomassie staining, 2 µg of the purified fusion proteins were denatured in Laemmli buffer, resolved by 8% SDS-PAGE and stained with Coomassie.

### Size-Exclusion Chromatography

Approx. 20 µg protein was applied to a BioSep-SEC-S2000 column (Phenomenex, Aschaffenburg, Germany) equilibrated with PBS and eluted at a flow rate of 0.5 ml/min using high-performance liquid chromatography (HPLC). For determining the size of recombinant proteins, standard proteins were run under the same conditions.

### TNFR-Binding Assay

ELISA plates (Greiner, Frickenhausen, Germany) were coated with mouse TNFR1-Fc or TNFR2-Fc fusion proteins at 1 µg/ml in PBS and incubated at 4°C overnight. Residual binding sites were blocked with 2% skim milk powder in PBS at RT for 2 h. TNF muteins were diluted in 2% skim milk powder in PBS and incubated for 1 h at RT. Bound proteins were detected with mouse monoclonal antibodies to TNF (clone HP8001; 1 µg/ml) or his-tag (Dianova; incubation for 1 h at RT) and HRP-conjugated anti-rabbit or anti-mouse IgG antibodies (diluted 1:10,000; incubation for 1 h at RT), followed by incubation with TMB substrate solution. Reaction was stopped by addition of 1 M H_2_SO_4_ and the absorbance at 450 nm was determined with an absorbance reader (Multiskan FC, Thermo Scientific, Karlsruhe, Germany) and data were analyzed using the software Microsoft Excel and GraphPad Prism 4 (GraphPad, La Jolla, CA). Between each step, nonbound proteins were removed by washing 4 times with 0.005% Tween-20 in PBS.

### Quartz Crystal Microbalance

Affinities of the fusion proteins for TNFR2 were determined by quartz crystal microbalance measurements (Attana Cell 200, Attana, Stockholm, Sweden). Therefore, TNFR2-Fc fusion proteins were chemically immobilized on a carboxyl sensor chip according to the manufacturer’s protocol at a high density (270 Hz). Binding experiments were performed in PBST (PBS, 0.1% Tween 20) pH 7.4 at a flow rate of 25 ml/min at 37°C. The chip was regenerated with 10 mM glycine HCl pH 2.0. Before each measurement, a baseline was measured which was subtracted from the binding curve. Data were collected using Attaché Office software (Attana, Stockholm, Sweden) and TraceDrawer (Ridgview Instruments, Vange, Sweden). K_d_ values were calculated from the measured association- and dissociation-rates (Kd = k_off_/k_on_) assuming a 1:1 binding stoichiometry.

### Cytotoxicity Assay

L929 cells (1.5 x 10^4^ cells/well) were grown in 96-well flat bottom cell culture plates overnight. L929 cells were treated with actinomycin D (1 µg/ml) for 30 minutes prior to addition of fusion proteins. Then, cells were incubated with different concentrations of the fusion proteins for 24 h at 37°C. Cells were washed with PBS and incubated with crystal violet (20% methanol; 0.5% crystal violet) for 20 minutes to stain viable cells. The dye was washed away under rinsing water and cells were air-dried. Crystal violet was resolved with methanol and the optical density at 550 nm was determined. Each sample was analyzed in triplicates and data were analyzed using the software Microsoft Excel and GraphPad Prism (GraphPad, La Jolla, CA).

### Enzyme-Linked Immunosorbent Assay

BV-2 cells were stimulated as indicated, supernatants were collected after 24 h and analyzed by an ELISA specific for Cxcl-2 (BV-2, R&D Systems, Minneapolis MN) according to the instructions of the manufacturer.

For determination of the inflammatory marker CRP in the blood, whole blood was withdrawn and analyzed by an ELISA specific for mouse CRP (R&D Systems, Minneapolis MN) according to the instructions of the manufacturer. IFNγ and IL-6 levels in the blood were determined by ELISAs specific for mouse IFNγ and IL-6 (Biolegend, Koblenz, Germany). The absorbance at 450 nm was determined and the amount of released Cxcl-2 or IL-6 was determined with the provided standard and calculated using the software GraphPad Prism.

### IL-2-Dependent Proliferation Assay

CT6 cells were cultivated with IL-2 (10 U/ml) for 2 weeks. Then, cells were harvested by centrifugation (300 g, 5 min) and washed twice with PBS (300 g, 5 min) to remove residual IL-2. To measure IL-2 induced proliferation, cells (1.5 x 10^4^ cells/well) were seeded in 96-well U form plates and incubated in presence of different concentrations of the fusion proteins for 48 h. Number of cells was determined by MTT (3-(4,5-Dimethylthiazol-2-yl)-2,5-Diphenyltetrazolium Bromide) assay. Therefore, cells were incubated with MTT (0.5 mg/ml) for 2 h at 37°C. Then, lysis buffer (10% SDS, 20 nM HCl) was added, cells were lyzed overnight and optical density at 550 nm was determined. Each sample was analyzed in triplicates and data were analyzed using the software Microsoft Excel and GraphPad Prism.

### Mouse T Cell Isolation and Culture

Spleens from C57BL/6 wildtype mice were dissociated through a 40 µm cell strainer and collected in 10 ml MACS buffer (PBS, 0.5% BSA, 2 mM EDTA). Splenocytes were centrifuged (300 g, 5 min) and washed once with 10 ml MACS buffer. Then CD3^+^ (purity > 98%) and CD4^+^ (purity > 96%) T cells were isolated using the FACS Aria III plated in αCD3-coated (6 h at 4°C) 96-well (U form) plates and cultivated in presence of soluble αCD28 and the fusion proteins. If indicated in the figure legend, cells were incubated with inhibitors for IL2R or TNFR2 before addition of fusion proteins. After 4 days, expression of IFNγ, CD25, and FoxP3 were determined by flow cytometry. For IFNγ staining, cells were reactivated with PMA/ionomycin in presence of brefeldin A for 4 h. For cytoplasmatic staining, the FoxP3 Staining Buffer Set (Miltenyi Biotech; Bergisch-Gladbach, Germany) was used according to the manufacturer’s instructions. Data were acquired using a MACSQuant Analyzer 10 (Miltenyi) and analyzed with FlowJo (FlowJo, LLC).

### RNA Isolation, cDNA Synthesis, and Quantitative Real-Time PCR

Total RNA was extracted using the PureLink RNA Mini Kit (ThermoFisher) and then transcribed into cDNA with the Maxima First Strand cDNA Synthesis Kit for RT-qPCR (ThermoFisher). The obtained cDNA was used to determine the *FOXP3* gene expression by quantitative real-time PCR (qPCR; CFX96, Biorad) using specific primers (FoxP3 fwd: 5’-GCCATGGCAATAGTTCCTTC-3’; FoxP3 rev: 5’- CGAACATGCGAGTAAACCAA-3’, GAPDH fwd: 5’-GTGGCAAAGTGGAGATTGTTGCC-3’; FoxP3 rev: 5’-GATGATGACCCGTTTGGCTCC-3’) and the Brilliant III Ultra-Fast SYBR Green QPCR Master Mix (Agilent, Waldbronn, Germany). To determine the expression of distinct genes, ΔΔCt values were determined by correlating the obtained values to the house-keeping gene *GAPDH*. Data are presented relative to the PBS controls as normalized fold expression.

### Statistics

Data are presented as mean ± standard error of the mean (SEM) of *n* independent experiments or animals. Normal distribution was analyzed by Shapiro-Wilk normality test. Statistical analyses were performed by Student’s t-test or analysis of variance, followed by a *post hoc* Tukey’s range test. A value of p < 0.05 was considered statistically significant.

## Results

### IL2-EHD2-sc-mTNF_R2_ Binds Selectively to TNFR2

**Figure 1:**
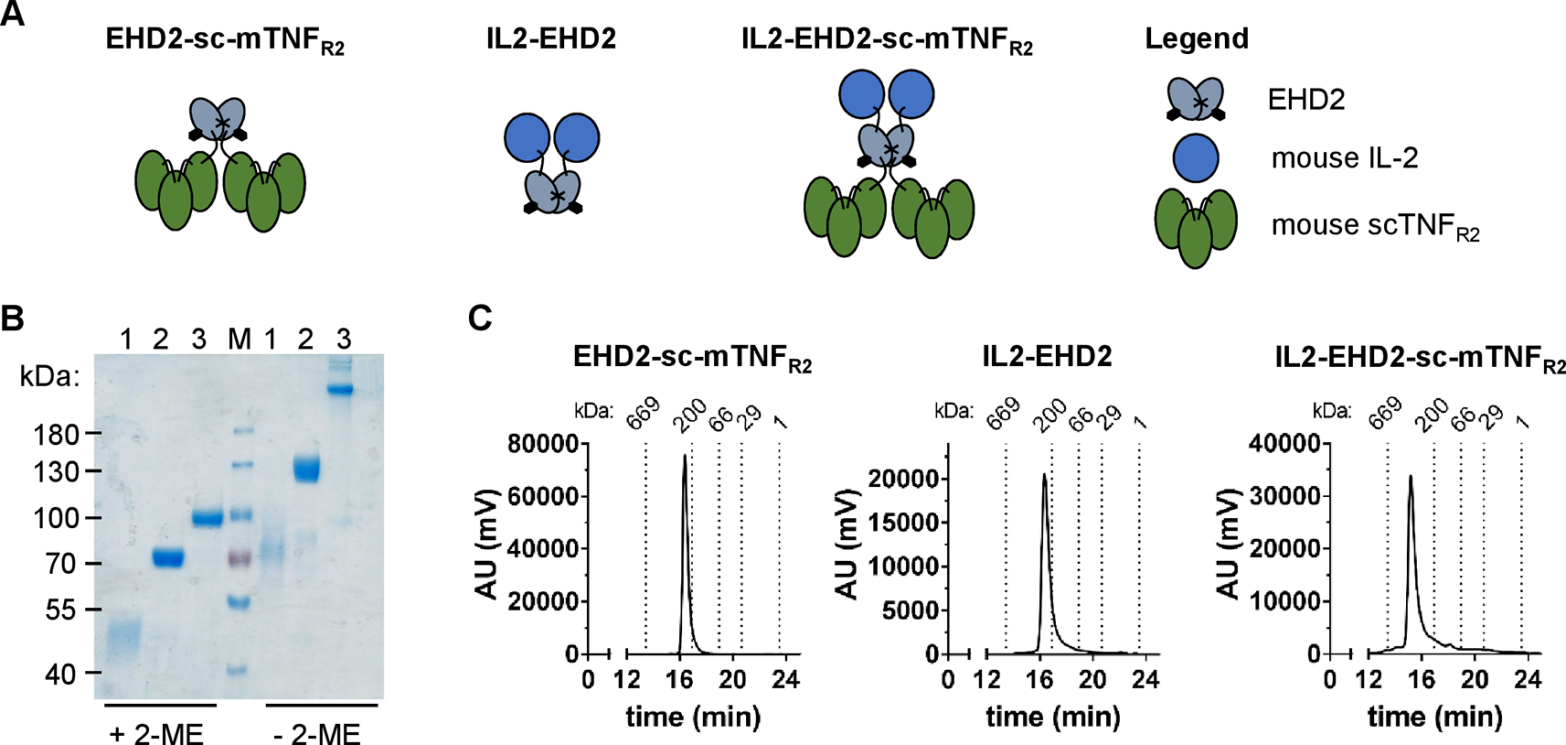
Biochemical characterization of fusion proteins. **(A)** Schematic representation and size-exclusion chromatography analysis of the different fusion proteins used in this study. **(B)** IL2-EHD2 (1), EHD2-sc-mTNF_R2_ (2), IL2-EHD2-sc-mTNF_R2_ (3), and a protein standard (M) were dissolved by SDS-Page under reducing (+2-ME) and nonreducing (-2-ME) conditions and proteins were visualized by Coomassie staining. **(C)** Size exclusion chromatography analysis of the different fusion proteins. Standard proteins of known molecular weight are indicated as dotted lines.

We previously demonstrated that oligomerized, covalently stabilized scTNF_R2_ fusion proteins mimic tmTNF and efficiently activate TNFR2 (Fischer et al., 2011; Dong et al., 2016; Fischer et al., 2017; Fischer et al., 2018). Our data indicate that these TNFR2 agonists expand Tregs *in vitro* and *in vivo* (Fischer et al., 2017; Fischer et al., 2018). *Ex vivo* studies with human and mouse T cells showed that the impact of TNFR2 activation on Treg proliferation is greatly enhanced in presence of IL-2 (Fischer et al., 2018). We therefore fused mouse IL-2 (aa 21-169) to the N-terminal end of the mouse TNFR2 agonist EHD2-sc-mTNF_R2_ to create a dimeric fusion protein (IL2-EHD2-sc-mTNF_R2_) that is capable of activating TNFR2 and IL-2 signaling at the same time. In all studies, IL2-EHD2-sc-mTNF_R2_ was compared to the fusion proteins IL2-EHD2 and EHD2-sc-mTNF_R2_ (**Figure 1A**), which are only able to activate IL-2 or TNFR2 signaling, respectively. All fusion proteins were expressed in HEK293-6E cells, isolated by IMAC in a single step using a N-terminal hexahistidyl-tag present in the molecules and further purified by preparative size-exclusion chromatography (SEC). Purity was confirmed by SDS-PAGE and Coomassie staining (**Figure 1B**). Under reducing conditions, the fusion proteins exhibited an apparent molecular mass of approximately 45 kDa (IL2-EHD2), 70 kDa (EHD2-sc-mTNF_R2_), and 90 kDa (IL2-EHD2-sc-mTNF_R2_), matching the calculated molecular mass of 32 kDa (IL2-EHD2), 68 kDa (EHD2-sc-mTNF_R2_), and 84 kD (IL2-EHD2-sc-mTNF_R2_). Under nonreducing conditions, a band of approximately 70 kDa (IL2-EHD2), 130 kDa (EHD2-sc-mTNF_R2_), and above 200 kD (IL2-EHD2-sc-mTNF_R2_) was observed, indicating the disulfide-stabilized dimer formation *via* the EHD2 domain. The oligomerization state of the fusion proteins was further confirmed by SEC (**Figure 1C**). All fusion proteins eluted as a single major peak, indicating the integrity and high purity of the molecules.

**Figure 2:**
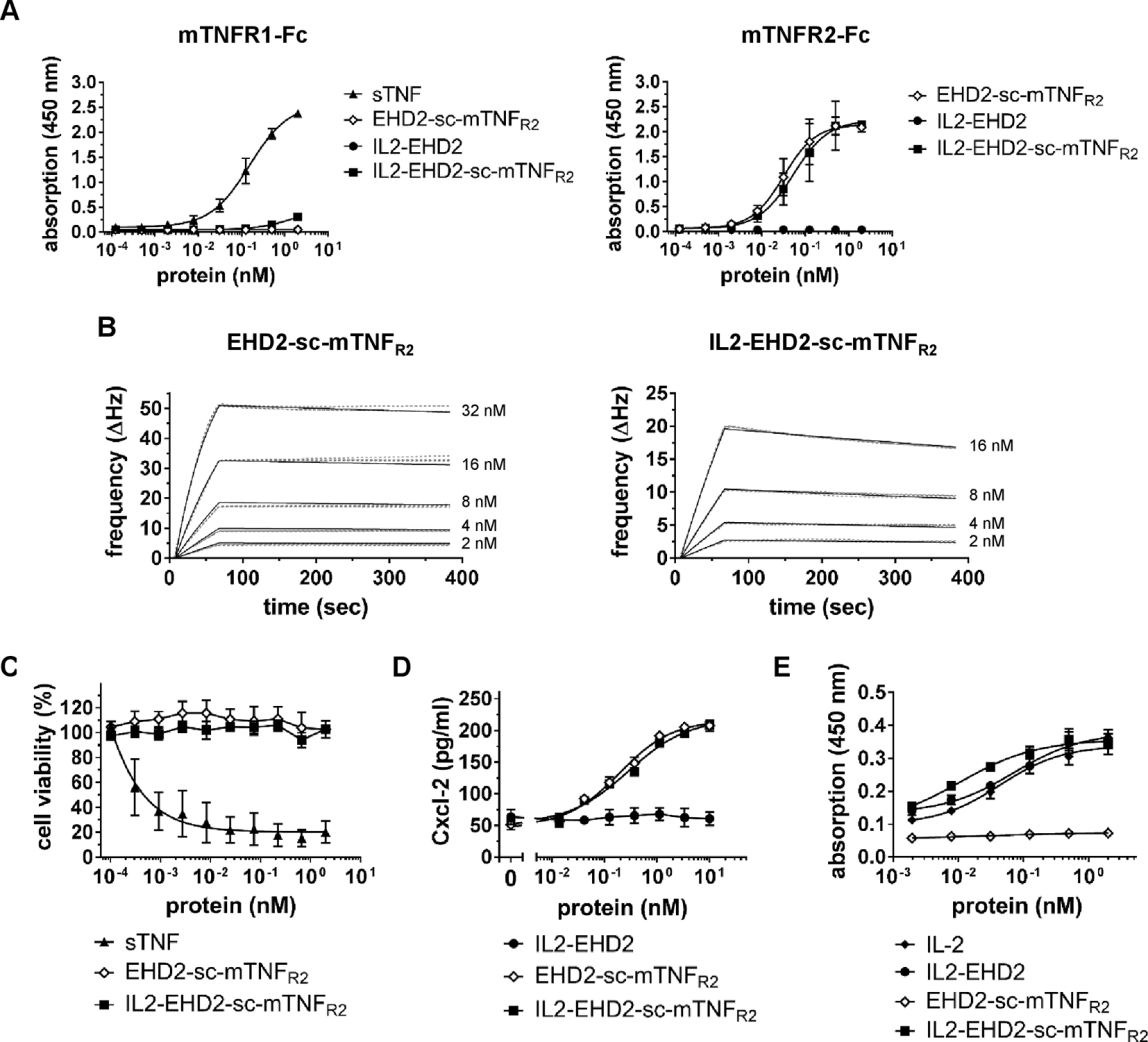
TNFR2-selective fusion proteins bind to TNFR2 with subnanomolar affinity. **(A)** Binding of fusion proteins to mouse TNFR1 and TNFR2 was analyzed by ELISA. Soluble recombinant mouse TNF (sTNF) was used as a positive control for binding to TNFR1 (n = 3 ± SEM). **(B)** Binding of TNF muteins to TNFR2 was analyzed by QCM using immobilized TNFR2-Fc. TNF muteins were analyzed at concentrations between 2 to 32 nM at 37°C in triplicates for each concentration (dashed lines = data curves, solid lines = fitted curves). **(C)** L929 cells were incubated with TNF muteins for 24 h. Soluble recombinant mouse TNF (sTNF) was used as a positive control for activation of TNFR1. Cell viability was measured using crystal violet staining (n = 3 ± SEM). **(D)** BV-2 cells were incubated for 24 h with the TNF muteins. Then supernatant was harvested and analyzed for presence of secreted Cxcl-2 by ELISA (n = 3 ± SEM). **(E)** CT-6 cells were incubated with fusion proteins and recombinant IL-2 for 48 h. Then, cell number was determined by measuring metabolic activity using the MTT assay (n = 9 ± SEM).

Binding of the fusion proteins to TNFR1 and TNFR2 was analyzed by ELISA using immobilized mouse TNFR1-Fc (mTNFR1-Fc) and mTNFR2-Fc fusion proteins. No binding to TNFR1 by EHD2-sc-mTNF_R2_ and IL2-EHD2 was observed at any tested concentration of the fusion proteins (**Figure 2A**). Unexpectedly, higher concentrations (above 1 nM) of IL2-EHD2-sc-mTNF_R2_ showed marginal interactions with the TNFR1-Fc coating (**Figure 2A**). However, the observed apparent binding at higher concentrations is likely irrelevant, since no bioactivity on TNFR1 was observed and the measurable bioactivity on TNFR2 was in the sub-nanomolar range (**Figure 2**). Both EHD2-sc-mTNF_R2_ (EC_50_ value of 0.031 nM) and IL2-EHD2-sc-mTNF_R2_ (EC_50_ value of 0.053 nM) bound to mTNFR2-Fc with similar binding efficacy (**Figure 2A**). As expected, IL2-EHD2 did not interact with TNFR1 or TNFR2 (**Figure 2A**). Next, we used quartz crystal microbalance measurements to determine the affinity of the fusion proteins to mTNFR2-Fc in a real-time binding analysis. Using a high-density chip (270 Hz) with saturated, immobilized TNFR2, we determined sub-nanomolar binding affinities of EHD2-sc-mTNF_R2_ and IL2-EHD2-sc-mTNF_R2_ (**Figure 2B**). IL2-EHD2-sc-mTNFR2 revealed a dissociation constant (K_d_) of 0.89 nM with an association rate constant k_on_ of 5.89×10^5^ M^−1^s^−1^ and a dissociation rate constant k_off_ of 4.77×10^-4^ M^−1^s^−1^. The K_d_ of EHD2-sc-mTNF_R2_ was approximately 7-times lower (0.12 nM) with a k_on_ of 1.2×10^6^ M^−1^s^−1^ and a k_off_ of 1.41×10^-4^ M^−1^s^−1^, indicating more stable binding of EHD2-sc-mTNF_R2_ than IL2-EHD2-sc-mTNF_R2_ to TNFR2. TNFR2-selectivity of the fusion proteins was confirmed using L929 cells. In contrast to wild-type soluble TNF (sTNF), which activates TNFR1 and induces cell death in L929 cells, none of the TNFR2-selective fusion proteins induced cell death in L929 cells (**Figure 2C**), verifying that fusion of IL2 to EHD2-sc-mTNF_R2_ did not impact TNFR2 selectivity of this novel molecule.

### IL2-EHD2-sc-mTNF_R2_ Efficiently Activates TNFR2 and IL2 Signaling

Knowing that fusion of IL-2 to EHD2-sc-mTNF_R2_ resulted in an increased off-rate of IL2-EHD2-sc-mTNF_R2_ to TNFR2-Fc, we investigated the potential impact on TNF and IL2 induced bioactivity of IL2-EHD2-sc-mTNF_R2_. Using TNFR2-dependent Cxcl-12 secretion in BV-2 cells as a readout, we showed that EHD2-sc-mTNF_R2_ and IL2-EHD2-sc-mTNF_R2_ possessed similar bioactivities, with EC_50_ values in the subnanomolar range (EHD2-sc-mTNF_R2_: 0.19 nM, IL2-EHD2-sc-mTNF_R2_: 0.28 nM) (**Figure 2D**). Bioactivity of the IL-2 component was evaluated using IL-2-dependent proliferation of CT-6 cells. Therefore, CT-6 cells were serum-starved and incubated in presence of recombinant IL-2, IL2-EHD2, EHD2-sc-mTNF_R2_, and IL2-EHD2-sc-mTNF_R2_. Proliferation rate was determined after 48 h. Whereas EHD2-sc-mTNF_R2_ did not impact proliferation of CT-6 cells as expected, IL-2 (EC_50_ value of 0.044 nM), IL2-EHD2 (EC_50_ value of 0.071 nM), and IL2-EHD2-sc-mTNF_R2_ (EC_50_ value of 0.01 nM) all increased proliferation with sub-nanomolar efficacy (**Figure 2E**).

### IL2-EHD2-sc-mTNF_R2_ Shows Superior Treg Expansion

**Figure 3:**
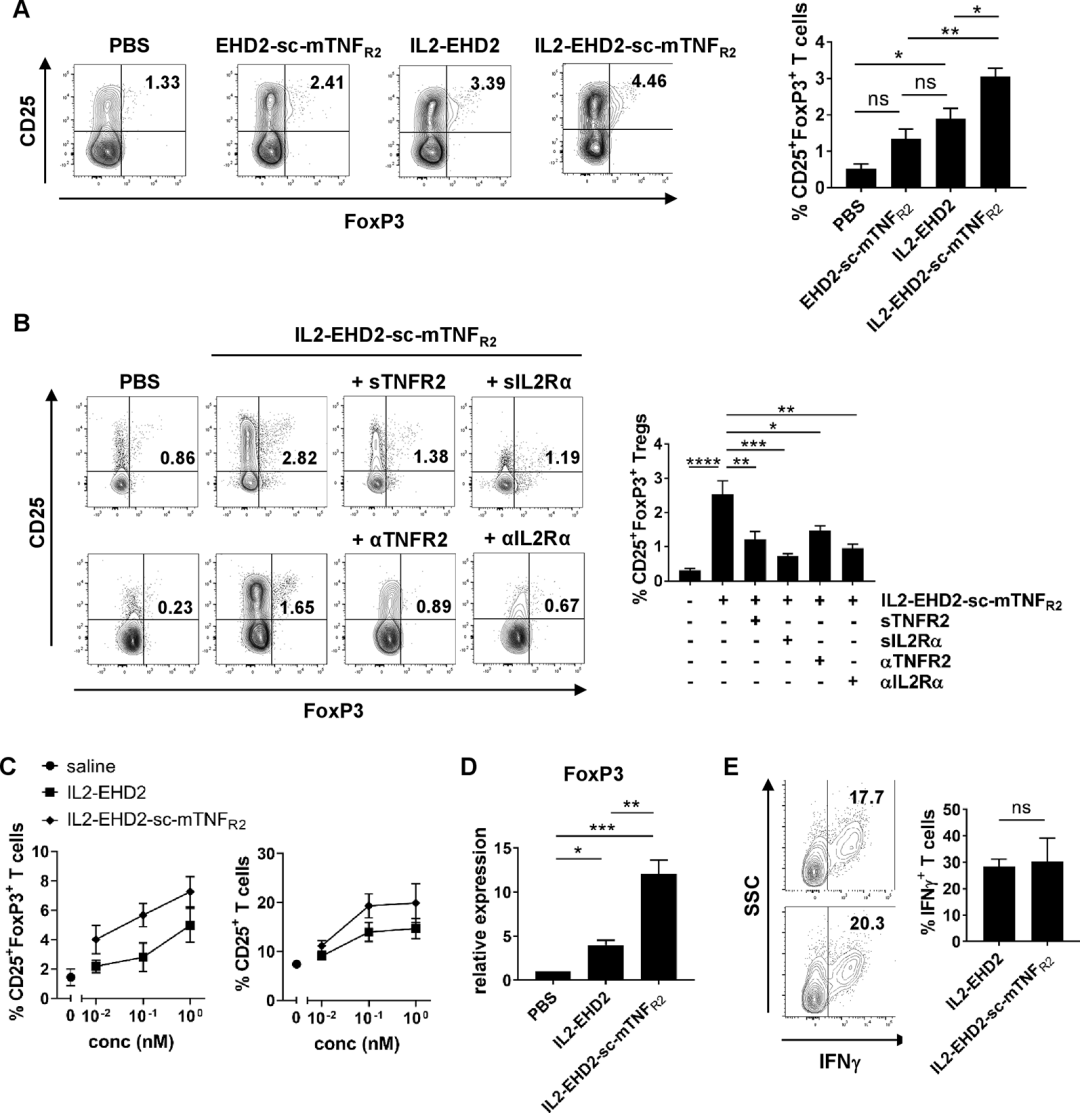
IL2 and sc-mTNF_R2_ domain are necessary for the bioactivity of IL2-EHD2-sc-mTNF_R2_ CD3^+^. T cells were isolated from mouse splenocytes and activated using plate-bound anti-CD3 (5 µg/ml) and soluble anti-CD28 (2 µg/ml). **(A)** Cells were cultivated in presence of 1 nM of the oligomerized muteins for 4 days. Percentage of CD25^+^FoxP3^+^ Tregs was determined by flow cytometry. **(B)** T cells were incubated for 30 min with either TNF (Enbrel, sTNFR2) or IL-2 (sIL2Rα) neutralizing reagents or antagonistic antibodies against TNFR2 (αTNFR2) or IL2Rα (αIL2Rα) before addition of IL2-EHD2-sc-mTNF_R2_ (1 nM). Then cells were cultivated for 4 days and percentage of CD25^+^FoxP3^+^ Tregs was quantified by flow cytometry. **(A**,**B)** Shown is a representative experiment (left panel). Combined data are from three independent experiments (right graph n = 3 ± SEM, one-way ANOVA). **(C)** Cells were incubated with different concentrations of the fusion proteins for 4 days. Percentage of CD25^+^ activated T cells and CD25^+^FoxP3^+^ Tregs was determined by flow cytometry (saline: n = 4, IL2-EHD2: n = 3, IL2-EHD2-sc-mTNF_R2_: n = 4, ± SEM). **(D)** Cells were incubated with 1 nM of the fusion proteins for 4 days, RNA was isolated, transcribed into cDNA and expression of *FOXP3* was analyzed by qPCR (n = 3 ± SEM, one-way ANOVA). **(E)** Cells were incubated with different concentrations of the fusion proteins for 4 days and reactivated with PMA/ionomycin in presence of brefeldin A. Percentage of IFNγ^+^ T effector cells in the FoxP3^-^ subpopulation was determined by flow cytometry (n = 3 ± SEM, t test). *p < 0.05, **p < 0.01, ***p < 0.001, ns, not significant.

Having demonstrated that both the IL-2- and TNFR2-selective component of IL2-EHD2-sc-mTNF_R_ was bioactive, we then went on to analyze whether the fusion of IL-2 to EHD2-sc-mTNF_R2_ improved the activity of IL2-EHD2-sc-mTNF_R2_ to expand Tregs. Therefore, we purified CD3^+^ T cells by FACS resulting in a fraction of approx. 1% CD25^+^FoxP3^+^ Tregs (data not shown). Then, cells were activated using anti-CD3/anti-CD28 antibodies and cultivated in presence of 1 nM of the fusion proteins, a concentration that efficiently induced IL-2 and TNFR2 signaling in previous assays. After 4 days incubation, CD25 and FoxP3 expression was analyzed by flow cytometry. Compared to PBS stimulation, IL2-EHD2 significantly increased the percentage of CD25^+^FoxP3^+^ Tregs within the population of CD3^+^ T cells, whereas IL2-EHD2-sc-mTNF_R2_ significantly increased the percentage of CD25^+^FoxP3^+^ Tregs compared to both EHD2-sc-mTNF_R2_ and IL2-EHD2 (**Figure 3A**), indicating the superior Treg expanding bioactivity of IL2-EHD2-sc-mTNF_R2_.

### IL2 and sc-mTNF_R2_ Components Are Necessary for Superior Treg Expansion by IL2-EHD2-sc-mTNF_R2_

After confirming that IL2-EHD2-sc-mTNF_R2_ expands Tregs more efficiently than EHD2-sc-mTNF_R2_ and IL2-EHD2, we went on to analyze whether both the IL2 and the sc-mTNF_R2_ component are necessary for the superior bioactivity of IL2-EHD2-sc-mTNF_R2_. Therefore, we activated purified CD3^+^ T cells using anti-CD3/anti-CD28 antibodies and cultivated the cells in presence of 1 nM IL2-EHD2-sc-mTNF_R2_ together with inhibitors of the TNF or IL-2 pathway. Inhibition of TNFR2 activation using neutralization of the sc-mTNF_R2_ moiety with soluble TNFR2 (sTNFR2) or blocking of TNFR2 with antagonistic antibodies against TNFR2 (αTNFR2) resulted in reduced IL2-EHD2-sc-mTNF_R2_-mediated Treg expansion (**Figure 3B**). Similar, inhibition of IL-2 signaling using neutralization of the IL2 domain with soluble IL-2 receptor α (sIL2Rα) or blocking of IL-2 receptor α with antagonistic antibodies against IL2R (αIL2Rα) resulted in reduced IL2-EHD2-sc-mTNF_R2_-mediated Treg expansion (**Figure 3B**), indicating that both the IL2 and the sc-mTNF_R2_ domain of IL2-EHD2-sc-mTNF_R2_ contribute to its increased bioactivity on immune cells.

To determine whether IL2-EHD2-sc-mTNF_R2_ may dose-dependent impact Tregs and T effector cells differently, we determined the fraction of CD25^+^ activated T cells and CD25^+^FoxP3^+^ Tregs after cultivation in presence of 0.01 nM, 0.1 nM, and 1 nM IL2-EHD2 or IL2-EHD2-sc-mTNF_R2_. Whereas IL2-EHD2-sc-mTNF_R2_ expanded Tregs more efficiently at all tested concentrations, the fraction of activated CD25^+^ T cells induced by IL2-EHD2 and IL2-EHD2-sc-mTNF_R2_ was similar at a concentration of 0.01 nM. In contrast, higher concentrations of IL2-EHD2-sc-mTNF_R2_ also induced T cell activation more efficiently (**Figure 3C**). We then confirmed that IL2-EHD2-sc-mTNF_R2_ expanded Tregs using a gene expression analysis. To this end, T cells were cultivated in presence of 1 nM of the fusion proteins for 4 days and gene expression of the Treg transcription factor FoxP3 was determined by quantitative real-time PCR. As expected incubation with IL2-EHD2-sc-mTNF_R2_ resulted in the highest *FOXP3* expression levels (**Figure 3D**), confirming the superior Treg expanding activity. We next investigated the impact of the fusion proteins on T effector cells using expression of the Th1 cytokine IFNγ as a read-out. Therefore, T cells were cultivated in presence of the fusion proteins for 4 days. Then, cells were reactivated using PMA/ionomycin in presence of brefeldin A and expression of IFNγ in the FoxP3**^-^** cell population was analyzed by flow cytometry. No differences in the expression of IFNγ between IL2-EHD2 and IL2-EHD2-sc-mTNF_R2_ cultivated cells were observed (**Figure 3E**).

### Phenotype of IL2-EHD2-sc-mTNF_R2_ Expanded Tregs

**Figure 4:**
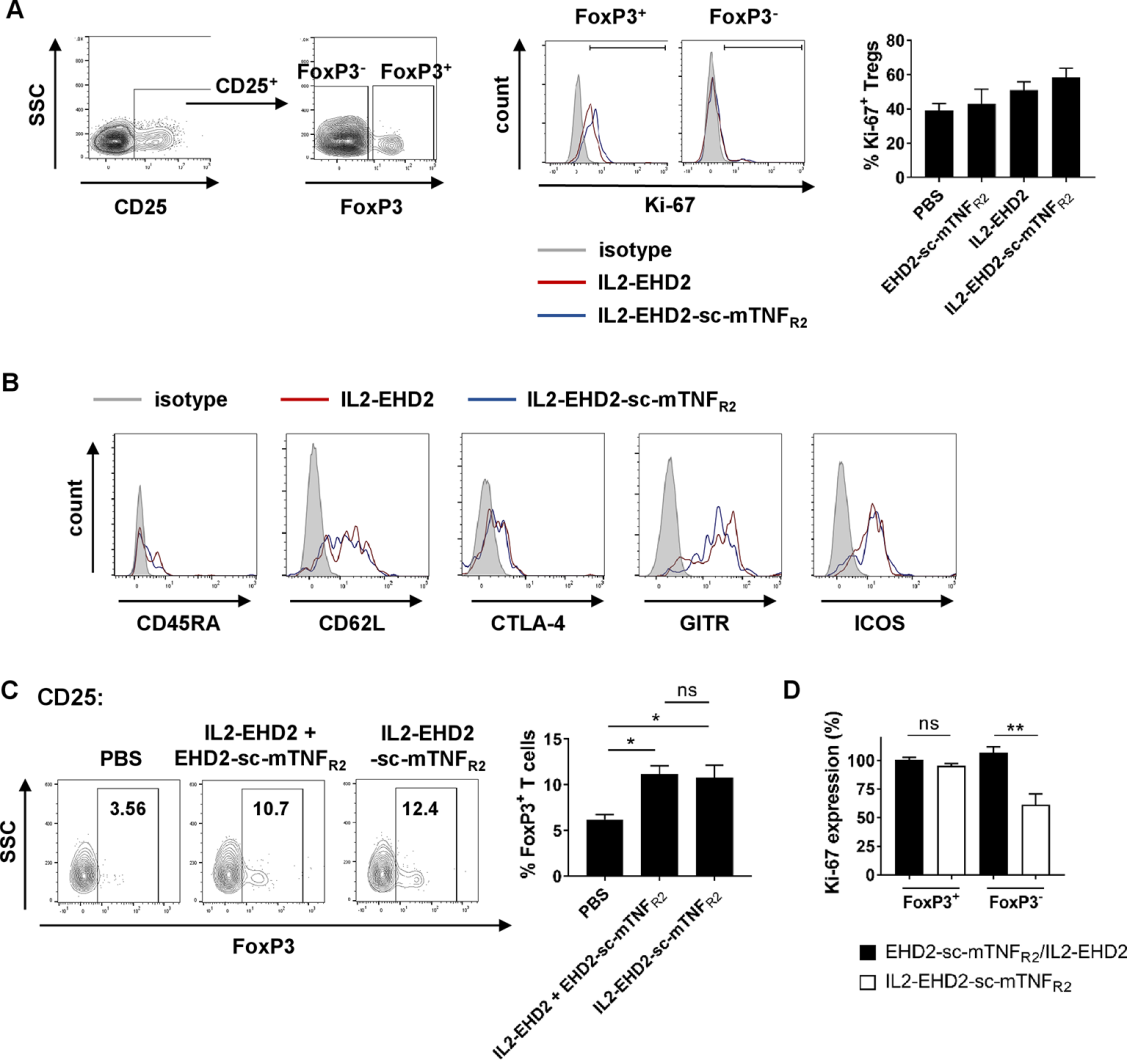
Phenotype of IL2-EHD2-sc-mTNF_R2_ expanded Tregs. CD4^+^ T cells were isolated from mouse splenocytes and activated using plate-bound anti-CD3 (5 µg/ml) and soluble anti-CD28 (2 µg/ml). **(A, B)** Cells were cultivated in presence of 1 nM of the oligomerized muteins for 4 days. **(A)** Cells were first gated for CD25^+^ cells and then percentage of proliferating Ki-67^+^ cells with the FoxP3^+^ and FoxP3^-^ cell population was determined by flow cytometry. Shown is a representative experiment (left panel) and quantification from three independent experiments ± SEM (analyzed by one-way ANOVA). **(B)** Expression of CD45RA, CD62L, CTLA-4, GITR, and ICOS was quantified in the CD25^+^ FoxP3^+^ subpopulation by flow cytometry. Shown are representative histograms from 3 independent experiments. **(C)** Cells were cultivated in presence of 1 nM IL2-EHD2-sc-mTNF_R2_ or the combination of IL2-EHD2 and EHD2-sc-mTNF_R2_ for 4 days. Percentage of FoxP3^+^ Tregs with the population of CD25^+^ cells was determined by flow cytometry. Shown is a representative experiment (left panel) and quantification from three independent experiments ± SEM (analyzed by one-way ANOVA). **(D)** Cells were cultivated in presence of 0.01 nM or 1 nM IL2-EHD2-sc-mTNF_R2_ or the combination of IL2-EHD2 and EHD2-sc-mTNF_R2_ for 4 days. Expression of Ki-67 was analyzed by flow cytometry. Results are presented as Ki-67 expression at a concentration of 0.01 nM relative to the Ki-67 expression at a concentration of 1 nM (n = 3 ± SEM, t test). *p < 0.05, **p < 0.01, ns, not significant.

Since TNFR2 mainly promotes expansion of CD4^+^ Tregs (Chen et al., 2007; Chen et al., 2010; Chen et al., 2013; Chopra et al., 2016; Mehta et al., 2018), we next investigated Treg expansion within the CD4^+^ T cell population. Analysis of IL-2/TNFR2-induced proliferation by Ki-67 staining demonstrated that all fusion proteins mainly induced proliferation of CD25^+^ FoxP3^+^ Tregs at a concentration of 1 nM (**Figure 4A**). Whereas CD25^+^ FoxP3^+^ Tregs showed a high percentage of Ki-67^+^ cells, only a low percentage of CD25^+^ FoxP3^-^ T effector cells were positive for Ki-67, indicating that the components induce expansion of Tregs. This is in line with published reports that TNFR2 activation induces expansion of natural Tregs and not induction of induced Tregs (Chopra et al., 2016). Even though IL2-EHD2-sc-mTNF_R2_ treatment resulted in the highest fraction of proliferating FoxP3^+^Ki-67^+^ Tregs the difference to EHD2-sc-mTNF_R2_ or IL2-EHD2 treatment was not significant (**Figure 4A**). We then characterized the phenotype of the expanded Tregs. IL2-sc-mTNF_R2_-expanded Tregs expressed CD45RA, CD62L, CTLA-4, GITR, and ICOS, surface proteins important for Treg function. However, no major differences in the expression levels of these proteins were observed between IL2-EHD2 and IL2-EHD2-sc-mTNF_R2_ expanded Tregs (**Figure 4B**).

Finally, we analyzed Treg expansion *via* IL2-EHD2-sc-mTNF_R2_ compared to combination of its building blocks, IL2-EHD2 and EHD2-sc-mTNF_R2_. At a concentration of 1 nM, both IL2-EHD2-sc-mTNF_R2_ and IL2-EHD2/EHD2-sc-mTNF_R2_ treatment induced similar expansion of CD25^+^ FoxP3^+^ Tregs, indicating that the genetic fusion of the single components does not result in improved bioactivity compared to the combination treatment (**Figure 4C**). We then compared the impact of 0.01 nM and 1 nM fusion protein on proliferation of FoxP3^+^ Tregs and FoxP3^-^ T cells. To this end, we calculated the Ki-67 expression at a concentration of 0.01 nM relative to the expression at 1 nM of the fusion proteins (**Figure 4D**). No differences in the expression of Ki-67 within the FoxP3^+^ Treg population were observed after stimulation with 0.01 nM and 1 nM of IL2-EHD2-sc-mTNF_R2_ and the combination of IL2-EHD2 and EHD2-sc-mTNF_R2_. Similar, stimulation with 0.01 nM or 1 nM IL2-EHD2 and EHD2-sc-mTNF_R2_ resulted in a comparable Ki-67 expression in the FoxP3^-^ T cell subpopulation. In contrast, 0.01 nM of IL2-EHD2-sc-mTNF_R2_ induced a significantly reduced Ki-67 expression comparted to a higher concentration of 1 nM. This suggests that while higher concentrations of IL2-EHD2-sc-mTNF_R2_ or the combination of IL2-EHD2 and EHD2-sc-mTNF_R2_ induce proliferation of FoxP3^-^ T effector cells with a similar efficacy, lower concentrations of IL2-EHD2-sc-mTNF_R2_ loose their activity on FoxP3^-^ T effector cells, while the combination of IL2-EHD2 and EHD2-sc-mTNF_R2_ still impact T effector cell proliferation like higher concentrations.

## Discussion

We and others have shown that exogenous TNFR2 activation promotes the expansion of Tregs *in vitro* and *in vivo* (Okubo et al., 2013; Chopra et al., 2016; Okubo et al., 2016; Fischer et al., 2017; Fischer et al., 2018; Lamontain et al., 2018) and that TNFR2 and IL-2 synergistically promote Treg expansion (Fischer et al., 2018). We therefore genetically fused mouse IL-2 to a TNFR2-selective TNF mutein resulting in IL2-EHD2-sc-mTNF_R2_, a fusion protein that efficiently activated both IL-2 and TNFR2 signaling. Our data show that IL2-EHD2-sc-mTNF_R2_ retained an affinity for TNFR2 in the sub-nanomolar range and efficiently activated TNFR2 and IL-2 signaling. IL2-EHD2-sc-mTNF_R2_ showed superior Treg-expanding activities compared to IL2-EHD2 and EHD2-sc-mTNF_R2_ stimulation alone, with both the IL2 and the EHD2-sc-mTNF_R2_ component necessary for the increased bioactivity. Altogether, our data indicate that IL2-EHD2-sc-mTNF_R2_ is a promising novel dual-acting cytokine fusion protein that might be superior to current Treg expanding strategies based on low-dose IL-2 monotherapy.

Tregs only constitute 1% to 2% of peripheral blood lymphocytes, but they are the master controllers of self-tolerance to avoid autoimmunity and guarantee long-term immune homeostasis (Sakaguchi et al., 2008). Accordingly, deregulation of Treg numbers or function have been implicated in the pathology of a variety of diseases. The most prominent example is the IPEX (immuno-dysregulation, polyendocrinopathy, enteropathy, X-linked) syndrome, a disease that develops due to mutations in the *FOXP3* gene leading to Treg defects and resulting in lethal multi-organ inflammation and autoimmunity (Bacchetta et al., 2018). Clinical studies of patients further revealed that there is a defect in either the number or the function of Treg cells isolated from the peripheral blood in various autoimmune disorders, such as type 1 diabetes, multiple sclerosis, systemic lupus erythematosus, myasthenia gravis and, rheumatoid arthritis (Miyara et al., 2011; Dominguez-Villar and Hafler, 2018). Thus, a number of ongoing clinical trials are repurposing approved drugs, such as the Treg growth factor IL-2 (aldesleukin) or Treg-stabilizing factors, e.g., the mTOR-inhibitor rapamycin to enhance Treg function as a therapeutic approach to control a variety of autoimmune diseases, GvHD, or organ transplant rejection (Abbas et al., 2018).

IL-2, which was first discovered as an autocrine growth factor for cultured T effector cells, also plays an important role for Treg survival and is essential for the functional capacity of Treg cells. IL-2 is required for the stable expression of Foxp3 (Fontenot et al., 2005; Feng et al., 2014) and other mediators of Treg cell–suppressive activity such as CD25 and CTLA-4 (Barron et al., 2010). Due to its parallel role for T effector cell function, administration of high concentrations of IL-2 cause toxicity, including vascular leak syndrome and other manifestations of a cytokine storm (Abbas et al., 2018). It is now well established that low-dose IL-2 therapy preferentially activates Tregs because of the constitutive high expression of CD25 as a subunit of the trimeric high affinity IL-2R (Klatzmann and Abbas, 2015; Abbas et al., 2018). Clinical trials have been conducted in hepatitis virus–associated vasculitis (Saadoun et al., 2011), chronic graft-versus-host disease (GVHD) (Koreth et al., 2011), systemic lupus erythematosus and type 1 diabetes (Klatzmann and Abbas, 2015). However, despite promising results, a continuing concern with this approach is that the therapeutic window for doses may be small, due to potential activation of the effector arm of the immune system, thus carrying the risk of exacerbating disease (Abbas et al., 2018). Our data indicate that IL2-EHD2-sc-mTNF_R2_ shows superior Treg-expanding bioactivity, compared to either IL2-EHD2 or EHD2-sc-mTNF_R2_ single treatments. Therefore, lower doses of this dual-acting cytokine may be therapeutic, without inducing toxic side effects, i.e., we hypothesize that IL2-EHD2-sc-mTNF_R2_ might have a larger therapeutic window than conventional low dose IL-2 protocols. However, at a first glance our *in vitro* data presented here suggest that costimulation with IL2-EHD2 and EHD2-sc-mTNF_R2_ together displays equal bioactivity as the fusion protein at concentrations of 1 nM. However, scrutinizing potential differences in dose response relationships between Treg and Teff, we noted that at low doses, the co-application of the single acting cytokines was superior in Teff expansion as compared to the dual action cytokine fusion protein. Therefore, we propose that the therapeutic window of the dual acting IL2-EHD2-sc-mTNF_R2_ fusion protein should be superior to treatment with IL-2 only or a combination of IL-2 with a monospecific TNFR2 agonist.

Other functions of IL-2 include its ability to inhibit the generation of proinflammatory T helper 17 cells (Laurence et al., 2007) and may synergize with its effects on Treg cells in the treatment of autoimmune and inflammatory diseases. Similar, next to its important role for expansion, function and stability of mouse and human Tregs (Chen et al., 2007; Chen et al., 2010; Nagar et al., 2010; Chen et al., 2013; Okubo et al., 2013), cell-intrinsic TNFR2 signaling has been shown to impair Th17 differentiation by promoting IL-2 expression (Miller et al., 2015). Further, TNFR2 has a well-documented role for tissue regeneration, i.e., TNFR2 promotes remyelination (Arnett et al., 2001), pancreatic regeneration (Ban et al., 2008; Faustman and Davis, 2013), and cardioprotection (Wang et al., 2008). In particular, TNFR2 was shown to selectively promote death of autoreactive T cells in human diabetes (Ban et al., 2008; Okubo et al., 2016) and promoting Treg activity is a promising approach to treat type I diabetes (Visperas and Vignali, 2016). Therefore, combining the bioactivity of IL-2 and a TNFR2 agonists in one molecule may be superior to conventional Treg expanding therapies due to (1) more efficient Treg expansion and (2) promotion of additional beneficial responses, such as tissue regeneration *via* the TNFR2-selective component.

In addition, efforts were described to engineer pharmacologically superior and Treg-selective IL-2 variants, e.g. by increasing the affinity of IL-2 for the α-chain (CD25) of the high-affinity-IL-2Rαβγ (Rao et al., 2005) or by decreasing the cytokines affinity to the β-chain in the intermediate affinity IL-2Rβγ (Peterson et al., 2018). As a future perspective, the combination of such a genetically optimized human IL-2 mutein with a human TNFR2-selective mutein (Fischer et al., 2011; Dong et al., 2016) might be a promising novel dual-acting drug prototype to promote immune tolerance and tissue regeneration.

## Data Availability Statement

All datasets generated for this study are included in the article/supplementary material.

## Author Contributions

TP, MS, CH, NP, and RF conducted experiments. JB, KP, and RK designed parts of the experimental work, analyzed the results and contributed to writing the manuscript. RF designed and supervised the project, analyzed the results and wrote the manuscript.

## Funding

RF was supported by the Carl Zeiss Foundation (Az. 0563-2.8./508/2) and a DFG research fellowship (FI 2138/1-1). The fusion proteins described in this work can be obtained *via* a Material Transfer Agreement (MTA).

## Conflict of Interest

RF and JB are named inventors on patent applications covering the clinical use of TNFR2 agonists. RF, RK, and KP are named inventors on patent applications covering the TNFR2 agonists technology.

The remaining authors declare that the research was conducted in the absence of any commercial or financial relationships that could be construed as a potential conflict of interest.
